# Epoxyeicosatrienoic Acids Inhibit the Activation of Murine Fibroblasts by Blocking the TGF-*β*1-Smad2/3 Signaling in a PPAR*γ*-Dependent Manner

**DOI:** 10.1155/2022/7265486

**Published:** 2022-10-13

**Authors:** Jia-Hao Tao, Tian Liu, Chen-Yu Zhang, Cheng Zu, Hui-Hui Yang, Yu-Biao Liu, Jin-Tong Yang, Yong Zhou, Cha-Xiang Guan

**Affiliations:** ^1^Department of Physiology, School of Basic Medicine Science, Central South University, Changsha, Hunan 410078, China; ^2^College of Physiology Education, Chongqing University of Arts and Science, Chongqing 412160, China

## Abstract

**Background:**

Epoxyeicosatrienoic acids (EETs), the metabolite of arachidonic acid by cytochrome P450 (CYP), reportedly serve as a vital endogenous protective factor in several chronic diseases. EETs are metabolized by soluble epoxide hydrolase (sEH). We have observed that prophylactic blocking sEH alleviates bleomycin- (BLM-) induced pulmonary fibrosis (PF) in mice. However, the underlying mechanism and therapeutic effects of EETs on PF remain elusive.

**Objective:**

In this study, we investigated the effect of CYP2J2/EETs on the activation of murine fibroblasts and their mechanisms.

**Results:**

we found that administration of the sEH inhibitor (TPPU) 7 days after the BLM injection also reversed the morphology changes and collagen deposition in the lungs of BLM-treated mice, attenuating PF. Fibroblast activation is regarded as a critical role of PF. Therefore, we investigated the effects of EETs on the proliferation and differentiation of murine fibroblasts. Results showed that the overexpression of CYP2J2 reduced the cell proliferation and the expressions of *α*-SMA and PCNA induced by transforming growth factor- (TGF-) *β*1 in murine fibroblasts. Then, we found that EETs inhibited the proliferation and differentiation of TGF-*β*1-treated-NIH3T3 cells and primary murine fibroblasts. Mechanistically, we found that 14,15-EET disrupted the phosphorylation of Smad2/3 murine fibroblasts by activating PPAR*γ*, which was completely abolished by a PPAR*γ* inhibitor GW9662.

**Conclusion:**

our study shows that EETs inhibit the activation of murine fibroblasts by blocking the TGF-*β*1-Smad2/3 signaling in a PPAR*γ*-dependent manner. Regulating CYP2J2-EET-sEH metabolic pathway may be a potential therapeutic option in PF.

## 1. Introduction

Pulmonary fibrosis (PF) is the buildup of chronic injury within the mesenchyma, and it represents the common final pathway of nearly all chronic and progressive lung diseases. It is characterized by inflammation, fibroblast proliferation, and differentiation with excessive collagen deposition [1]. IPF patients usually have progressive dyspnea and worsening pulmonary function, with a median survival of only 2–4 years after diagnosis [2]. Available treatments to slow PF progression and prevent it are pretty limited. The in-depth investigation of the pathological mechanism of PF and the search for potential therapeutic targets are imminent. The activation of fibroblasts is the central mechanism of PF, and transforming growth factor *β*1 (TGF-*β*1) plays a vital role. The TGF-*β*1/Smad signaling pathway is activated by oxidative stress and inflammation, characterized by an increased expression of TGF-*β*1 and phosphorylation of Smad2/3 [3]. Inactivation of fibroblast promises to alleviate or reverse PF [4–6].

Arachidonic acid (ARA) is a polyunsaturated fatty acid released by phospholipases. Our previous work has shown that ARA metabolism is disrupted in a bleomycin- (BLM-) induced murine PF model [7]. Epoxyeicosatrienoic acids (EETs) are synthesized from ARA by the cytochrome P450 (CYP), such as CYP2C and 2J families [8]. There are four isomerides, including 5,6-, 8,9-, 11,12-, and 14,15-EET [9]. CYP2J2 is the main enzyme mediating EET production in humans, mainly 14,15-EET [10]. EETs serve as a vital endogenous protective factor in various diseases [11–14]. EETs have been shown to have various biological effects, such as anti-inflammation, antiapoptosis, proangiogenesis, antioxidation, and antihypertension [15, 16]. EETs are metabolized by soluble epoxide hydrolase (sEH) rapidly. Inhibiting sEH has been shown to elevate the biological activity of EETs [17]. Our previous works have firstly demonstrated that protective inhibiting EET degradation with an sEH inhibitor [1-trifluoromethoxyphenyl-3-(1-propionylpiperidin-4-yl) urea (TPPU)] prevents BLM-induced PF in mice [18]. Other studies have also reported this phenomenon [19, 20]. However, the exact underlying mechanism remains unknown. Recently, it has been reported that 11,12-EET and sEH inhibition may prevent IPF by inhibiting the activation of patients' fibroblasts [20], while the effects and specific mechanisms of the CYP2J2-EETs-sEH pathway on the fibroblast activation during PF have not been fully elucidated.

EETs are natural ligands for peroxisome proliferation-activated receptor *γ* (PPAR*γ*) [21]. PPAR*γ* is a ligand-activated transcription factor, significantly beneficial in a series of chronic diseases, including cardiac remodeling [22] and renal fibrosis [23]. In vascular smooth muscle cells (VSMCs), PPAR*γ* inhibits metabolic and proliferative events by combining Smad3 directly and downregulating the TGF-*β*1/Smad3 signaling [24]. Nonetheless, the mechanisms of PPAR*γ* in EETs attenuating the activation of lung fibroblasts in PF are unclear.

In this study, we found that EETs therapeutically reversed PF by attenuating fibroblast activation in BLM-treated mice via PPAR*γ*. We showed that inhibition of sEH reversed lung fibrosis in BLM-treated mice. The overexpression of CYP2J2 and EETs could attenuate TGF-*β*1-induced activation of fibroblasts by suppressing Smad2/3 phosphorylation in a PPAR*γ*-dependent manner.

## 2. Materials and Methods

### 2.1. Animal

The male C57BL/6 mice (8 to10 weeks of age, 20-25 g) were provided by Hunan SJA Laboratory Animal Co., Ltd. (Hunan, China). They were feeding under pathogen-free conditions (12 h-dark/light cycle and free access to water and food). The experimental use of mice in the present study was approved by the Ethics Committee of the Institute of Clinical Pharmacology at Central South University (No. 2020ysdw0685) to keep with the guidelines of the National Institutes of Health.

### 2.2. BLM-Induced PF Model and Animal Treatment

Mice were randomly divided into the following groups using the table of random numbers: (i) the control group: intratracheal saline plus saline intraperitoneal, (ii) BLM group: intratracheal injection of BLM (1.5 mg/kg, in 50 *μ*L saline, Nippon Kayaku, Japan) plus an intraperitoneal injection of saline, and (iii) BLM + TPPU group: intratracheal BLM plus TPPU intraperitoneally (1 mg/kg/day). All treatment was under sodium pentobarbital (80 mg/kg) anesthesia. Each group includes at least 6 mice. TPPU dissolved in 0.1% ethanol was administered to mice daily from the 7^th^ to the 21^st^ day after BLM injection. Mice were humanely euthanized by sodium pentobarbital anesthesia on the 21^st^ day. The lungs and bronchoalveolar lavage fluid (BALF) were collected. Bodyweight was recorded throughout the experiment.

### 2.3. Collection of BALF

BALF was collected through intratracheal instillation with 0.8 mL saline at 4°C and repeated thrice. Total protein content in BALF, centrifuged at 1,500 rpm for 5 min, was detected by the BCA kit (Thermo Fisher Scientific, USA).

### 2.4. Lactate Dehydrogenase Assay

According to the instruction, the lactate dehydrogenase (LDH) activity in the BALF of mice was measured by the corresponding kits (Jiancheng Bioengineering Institute, China). The activity of LDH was determined by the optical density (450 nm).

### 2.5. Pulmonary Histopathology Analysis

Samples of the lungs taken from mice were used for histopathology analysis. Formalin-fixed (10%) paraffin-embedded tissue was sectioned at 3 *μ*m. Sections were stained with H&E to observe the pulmonary histopathology changes. Masson's trichrome stain was used to observe and assess collagen deposition. Slides were randomized, read blindly, and examined for percentage lung pathology.

### 2.6. Ashcroft Score

The severity of fibrotic changes in each histological section of the lung was assessed as the mean severity score from observed microscopic fields. Thirty fields in each section were analyzed. Grading was done in a blinded fashion by two observers, and the mean was taken as the fibrosis score [7].

### 2.7. Immunofluorescent Staining

For *α*-SMA staining, the following unstained slides were deparaffinized through standard methods. 3% H_2_O_2_ was used to inactivate any endogenous peroxidase for 30 min. The lung slices were blocked in Tris-buffered saline (TBS) with 5% bovine serum albumin (BSA, Solarbio, China) for 1 h. Then, slides were incubated with *α*-SMA antibody (1: 200, SAB) at 4°C overnight. Slides were washed with 1× TBS and then incubated with Goat Anti-Rabbit IgG H&L (1: 2000; Abcam) for 30 min. The nuclei were counterstained with 4,6-diamidino-2-phenylindole (DAPI, Invitrogen, USA). After washing the slides with PBS 3 times, slides were then mounted with 90% glycerol and detected by a fluorescence microscope (Nikon, Japan).

For PPAR*γ* staining, the samples were fixed with PFA and blocked with 5% BSA supplemented with 0.4% Triton-X for 1 h at room temperature. Fixed samples were incubated with the primary PPAR*γ* antibody (Sigma-Aldrich, 1 : 200) at 4°C overnight. After washing, the samples were incubated with the Goat Anti-Rabbit IgG H&L (1: 2000; Abcam) for 30 min and counterstained with DAPI. After mounting, the samples were detected by a fluorescence microscope (Nikon TS2R, emission spectrum 488 nm, absorption spectrum 519 nm).

### 2.8. Isolation of Primary Murine Lung Fibroblasts

Primary murine lung fibroblasts were isolated from lung tissue derived from 6-8-week-old C57BL/6 mice under sterile conditions. The mice were anesthetized with sodium pentobarbital and sacrificed. A small incision was cut on the right auricle after exposing the thorax. The lungs were operated on with pulmonary artery lavage from ventriculus sinister with sterile PBS (5 mL) three times. Subsequently, the lung tissue was cut into 1 mm diameter pieces by ophthalmic scissors, digested with 1 mg/mL collagenase I (Sigma-Aldrich, USA), and incubated at 37°C for 1 h. Afterward, cells were filtered by a 70 *μ*m cell strainer and grown in Dulbecco's Modified Eagle Medium/F12 (DMEM/F-12, Gibco, USA) containing 15% fetal bovine serum (FBS, Gibco), 100 U/mL penicillin, and 100 *μ*g/mL streptomycin.

### 2.9. Cell Treatment

Murine fibroblast NIH3T3 cell lines were purchased from the American Type Culture Collection (ATCC; CRL-1658). The cells were maintained in Dulbecco's Modified Eagle's Medium (DMEM, Gibco) supplemented with 10% bovine calf serum (BCS, Gibco) at 37°C and 5% CO_2_. The cells were seeded in 6-well plates. The next day, fibroblasts were starved (without serum) for 12 h and then treated with different compounds. To elevate the role of EETs in fibroblast activatioin, we constructed an adenovirus-CYP2J2. Cells were infected with adenovirus-CYP2J2 (1 × 10^9^/mL) or vector (1 × 10^9^/mL) with the complete growth medium for 48 h and then incubated with or without TGF-*β*1 (5 or 10 ng/mL, Sino Biological, China). Fibroblasts were exposed to 5,6-EET, 8,9-EET, 11,12-EET, 14,15-EET (0.1, 0.5, 1 *μ*M, Cayman, USA), or ethyl alcohol (1 : 300) for 5 min and then treated with TGF-*β*1 (5, 10 ng/mL) or vehicle control for 0.5 h to activate Smad2/3 signaling, for 24 h to active the p-PPAR*γ*, or for 48 h to induce the proliferation and differentiation. The cells were exposed to GW9662 (a PPAR*γ* inhibitor, 10 *μ*M, Merck, USA) or dimethyl sulfoxide (DMSO, Sigma-Aldrich) for 1 h with the following treatment of 14,15-EET and TGF-*β*1.

### 2.10. Cell Growth Assay

Cell Counting Kit-8 (CCK-8, Dojindo Molecular Technologies, Japan) was used to determine cell viability. Briefly, the cells (1000 cells/well) were seeded in 96-well plates. The next day, fibroblasts were starved (without serum) for 12 h and then treated with different compounds. Then, fibroblasts were exposed to 5,6-EET, 8,9-EET, 11,12-EET, 14,15-EET (0.1, 0.5, and 1 *μ*M, Cayman, USA), or ethyl alcohol (1 : 300) for 5 min and then treated with TGF-*β*1 (5, 10 ng/mL) or vehicle control for 48 h. Ten microliters of CCK-8 was added to each well, followed by incubation at 37°C for an additional 1 h. The absorbance was measured at 450 nm with an ultraviolet spectrophotometer (Thermo Fisher Scientific).

### 2.11. Cell Cycle Analysis

Flow cytometry was used to assay the cell cycle of murine fibroblast NIH3T3. Fibroblasts were starved for 24 h. The next day, the cells were maintained in no-serum DMEM with 14,15-EET (1 *μ*M) for 5 min before TGF-*β*1 (10 ng/mL) treatment for 24 h and harvested. The cells were digested by trypsin and centrifuged at 800 rpm for 5 min. After washing with PBS, the cells were resuspended in 70% ethanol at 4°C gently and fixed at 4°C overnight. The cells were centrifuged at 2000 rpm for 5 min and added to a staining buffer containing RNase A (50 *μ*g/mL, Solarbio, China) and at 4°C for 30 min before adding PI (0.25 mg/mL, Solarbio) to stain at 4°C for 30 min. The DNA profiles were determined by a flow cytometer (BD, USA).

### 2.12. Total RNA Extraction and PCR Analysis

The total RNA extraction was performed using RNAiso Plus (Takara Bio Inc., Kusatsu, Japan). RNA concentration and purity were determined using an ultraviolet spectrophotometer (Thermo Fisher Scientific, USA) at an absorbance ratio of 260 and 280 nm. cDNA synthesis was carried out using Prime Script RT Reagent Kit with gDNA Eraser (Takara Bio Inc.) according to our previous study [25]. The PCR amplifications were performed on the cDNA (1 *μ*L) in the presence of forward and reversed primers (10 *μ*M) and MIX (Takara Bio Inc.), followed by an initial incubation for 3 min at 94°C, 30 cycles of 30 s at 94°C, 30 s at 59°C, and 4 min at 70°C. The sequences of the specific primers are shown in Table 1. Data were digitized and analyzed by the Image Lab Analyzer software (Bio-Rad, CA, USA).

### 2.13. Protein Extraction and Western Blot

Total protein lysates were prepared using RIPA buffer (Solarbio, China) according to our previous study [26]. The cells were homogenized in RIPA buffer containing the cocktail (Solarbio) of protease inhibitors and stored at -80°C. The protein concentration was determined with Pierce™ BCA Protein Assay Kit (Thermo Fisher Scientific). Protein samples were dissolved in 2% SDS and denatured at 100°C for10 min. For SDS-PAGE, protein (30 *μ*g/well) from homogenate fractions was separated in 12% SDS-PAGE gels and transferred to 0.45 *μ*m polyvinylidene difluoride membranes. Membranes were blocked using 5% BSA at room temperature for 1 h. Antibodies against *α*-SMA (SAB, 1 : 3000), PCNA (Proteintech, 1 : 3000), *β*-tubulin (Proteintech, 1 : 2000), COL-I (CST, 1 : 1000), COL-III (SAB, 1 : 1000), Smad2 (Abcam, 1 : 2000), Smad3 (Abcam, 1 : 2000), p-Smad2 (Abcam, 1 : 2000), p-Smad3(Abcam, 1 : 2000), PPAR*γ* (Sigma-Aldrich, 1 : 1000), and p-PPAR*γ* (Invitrogen, 1 : 2000) were used at 4°C overnight. After being washed with TBST, the membrane was incubated with a horseradish peroxidase-conjugated secondary antibody at room temperature for 1 h and revealed by chemiluminescence (Millipore, USA). Images were captured with ChemiDoc XRS+ (Bio-Rad, CA). The protein and phosphorylated protein relative band intensity compared with *β*-tubulin or total protein was measured with the Image Lab Analyzer software (Bio-Rad, CA).

### 2.14. Statistical Analysis

Statistical analysis was performed with SPSS 22.0 and Graph Pad Prism software (Version 7.0, CA). Results are expressed as means ± SD. One-way analysis of variance (ANOVA) was used for multiple-group comparisons. Differences between the two groups were determined by a *t*-test. *P* < 0.05 was considered statistically significant.

## 3. Results

### 3.1. Inhibition of sEH Attenuates the PF Induced by BLM in Mice

**Figure 1:**
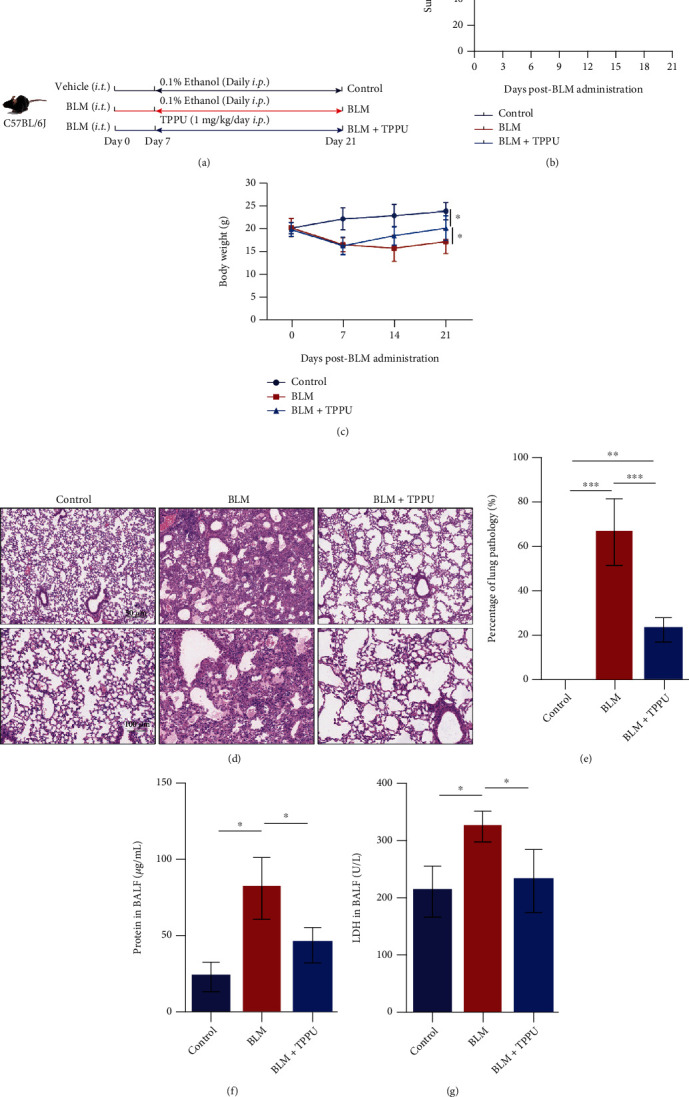
Inhibition of sEH attenuates the PF induced by BLM in mice. (a) TPPU (1 mg/kg/day, *i.p.*) was administered daily from the 7th day after BLM injection (1.5 mg/kg, *i.t.*). (b) Survival curves of mice (*n* = 10). (c) Bodyweight changes of mice (*n* = 6). (d) Lung sections of mice were analyzed by H&E staining. (e) The fibrotic area was expressed as a percentage of lung pathology (*n* =6). (f) The total protein concentration in BALF (*n* =6). (g) The LDH activity in BALF (*n* =6). ^∗^*P* < 0.05, ^∗∗^*P* < 0.01, and ^∗∗∗^*P* < 0.001.

To investigate whether inhibiting EET degradation affected PF, an sEH inhibitor, TPPU, was used on the 7^th^ day after BLM administration (Figure 1(a)). Similar to our previous study in which TPPU was used from the 1^st^ day after BLM injection [18], we found that TPPU treatment significantly increased the survival rate of mice compared with the BLM group (Figure 1(b)). Besides, TPPU reduced the weight loss of mice treated with BLM (Figure 1(c)). H&E stain demonstrated that TPPU therapeutically attenuated the percentage of average lung pathology in the lung after BLM exposure (Figures 1(d) and 1(e)). The total protein in BALF reflects pulmonary vascular permeability. Besides, LDH in BALF is positively related to cellular damage. We found that TPPU treatment robustly decreased the total protein content in BALF of BLM-treated mice (Figure 1(f)) and partially reduced the LDH activity (Figure 1(g)). Taken together, these results indicate that therapeutical inhibition of sEH attenuates fibrotic changes and lung injury in BLM-treated mice.

### 3.2. Inhibition of sEH Reverses Collagen Deposition in BLM-Treated Mice

**Figure 2:**
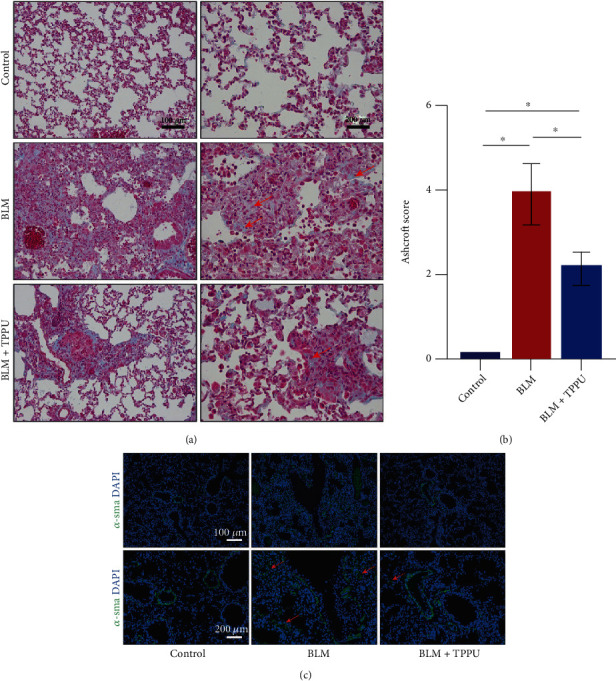
Inhibition of sEH reverses collagen deposition in BLM-treated mice. (a) Masson's trichrome staining in lung sections of BLM-treated mice. (b) The Ashcroft score was evaluated by three blinded pathologists (*n* = 6). (c) Representative results from lung sections stained with immunofluorescent staining for *α*-SMA. ^∗^*P* < 0.05.

The induction of matrix-producing myofibroblasts and excessive extracellular matrix accumulation is the characteristic of PF. We found that TPPU treatment significantly reduced the collagen disposition detected with the Masson stain (Figures 2(a) and 2(b)). We also found that TPPU reduced the fluorescence intensity of *α*-SMA in the lung compared to the BLM group (Figure 2(c)). These data indicate that therapeutically inhibiting sEH reverses collagen deposition in BLM-treated mice.

### 3.3. CYP2J2 Overexpression Attenuates TGF-*β*1-Induced Activation of Murine Fibroblasts

**Figure 3:**
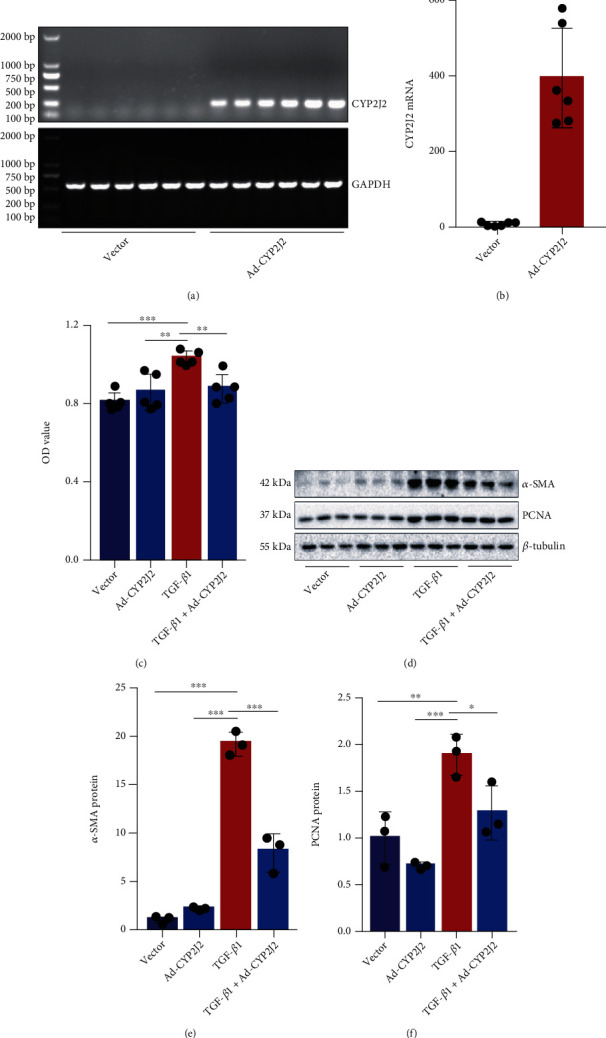
The CYP2J2 overexpression attenuates TGF-*β*1-induced activation of murine fibroblasts. The expression of *CYP2J2* mRNA in NIH3T3 cells after the adenovirus-CYP2J2 infection for 48 h was detected by PCR ((a, b), *n* = 5). The cell proliferation was examined by CCK-8 ((c), *n* = 5). Western blot was applied to detect the protein level of *α*-SMA and PCNA in NIH3T3 cells from each group ((d)–(f)(d)–(f), *n* = 3). ^∗^*P* < 0.05, ^∗∗^*P* < 0.01, and ^∗∗∗^*P* < 0.001.

To increase the EETs in fibroblasts, we used adenovirus-CYP2J2 to infect NIH3T3 cells. PCR results showed that *CYP2J2* mRNA was successfully overexpressed in NIH3T3 cells (Figures 3(a) and 3(b)). To validate the idea that endogenous elevating EETs decrease the activation of fibroblasts induced by TGF-*β*1, the proliferation and the differentiation of the cells were measured in the following study. Results showed that the CYP2J2 overexpression resulted in a significant reduction of proliferation induced by TGF-*β*1 (10 ng/mL) in NIH3T3 cells (Figure 3(c)). Besides, the western blot revealed that the CYP2J2 overexpression downregulated *α*-SMA and PCNA expression in NIH3T3 cells (Figures 3(d)–3(f)). These data indicate that the CYP2J2 overexpression attenuates the activation of murine fibroblasts induced by TGF-*β*1.

### 3.4. EETs Attenuate TGF-*β*1-Induced Activation of Murine Fibroblasts

**Figure 4:**
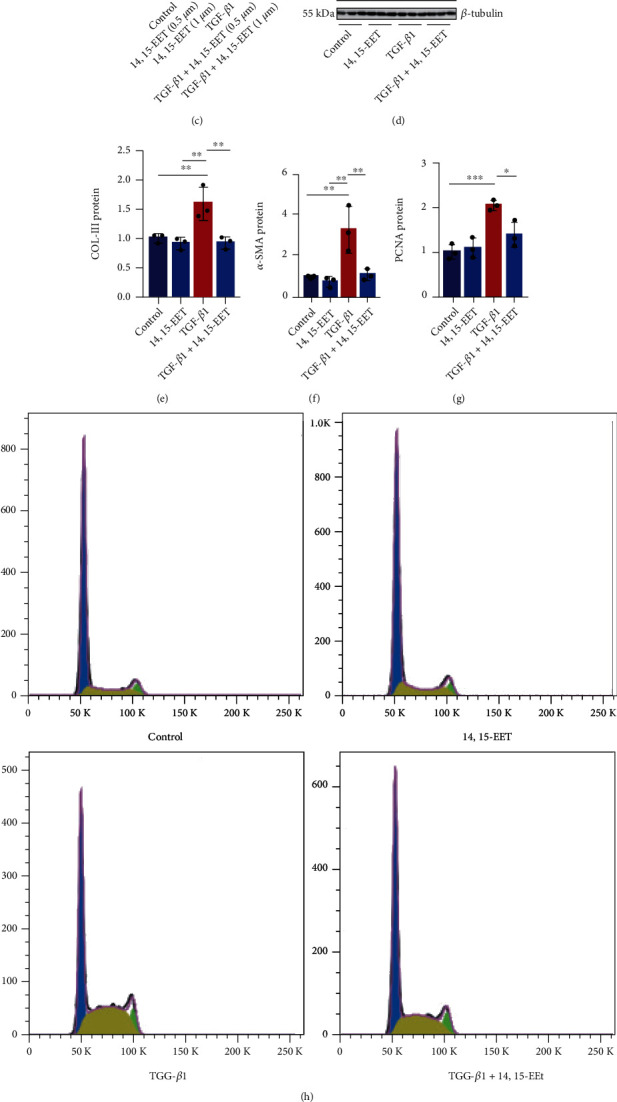
EETs attenuate TGF-*β*1-induced activation of murine fibroblasts. NIH3T3 cells were treated with 14,15-EET (1 *μ*M) for 5 min, followed by treatment with TGF-*β*1 (10 ng/mL) for 48 h. The cell proliferation was examined by CCK-8 ((a)–(c), *n* = 5). Western blot was applied to detect the protein level of COL-III, *α*-SMA, and PCNA in NIH3T3 cells from each group ((d)–(g), *n* = 3). Cell cycles were detected using flow cytometry ((h)–(j), *n* = 3). ^∗^*P* < 0.05, ^∗∗^*P* < 0.01, and ^∗∗∗^*P* < 0.001. ###*P* < 0.001 vs. TGF-*β*1 group.

To gain further insights into whether EETs reduced the activation of murine fibroblasts, we treated NIH3T3 cells with 5,6-EET, 8,9-EET, 11,12-EET, or 14,15-EET (0.1, 0.5, or 1 *μ*M) for 48 h. We found that EETs did not affect the proliferation of NIH3T3 cells without TGF-*β*1 stimulation (Figure 4(a)). While four regioisomers of EETs (1 *μ*M) observably inhibited the proliferation of NIH3T3 cells induced by TGF-*β*1. 14,15-EET was the most effective type compared to the other three types of EETs (Figure 4(b)). Therefore, we used 14,15-EET in the following experiments. CCK8 assay showed that 14,15-EET (0.5 or 1 *μ*M) inhibited the TGF-*β*1-induced proliferation of both NIH3T3 cells (Figure 4(c)) and primary murine lung fibroblasts (Figure S1A). Two types of cells were treated with 14,15-EET (1 *μ*M) and stimulated by TGF-*β*1 for 48 h. We found that 14,15-EET decreased the expressions of collagen, *α*-SMA, and PCNA protein of NIH3T3 cells (Figures 4(d)–4(g)) and primary murine lung fibroblasts induced by TGF-*β*1 (Figure S1B-E). Next, we use flow cytometry to assay the cell cycle of murine fibroblasts. The results showed that 14,15-EET significantly reduced the G0/G1 phase of the TGF-*β*1-treated NIH3T3 cells (Figures 4(h)–4(j)). These results suggest that EETs depress the activation and collagen synthesis of murine fibroblasts induced by TGF-*β*1.

### 3.5. 14,15-EET Disrupts the TGF-*β*1-Smad2/3 Signaling in Murine Fibroblasts by Activating PPAR*γ*

**Figure 5:**
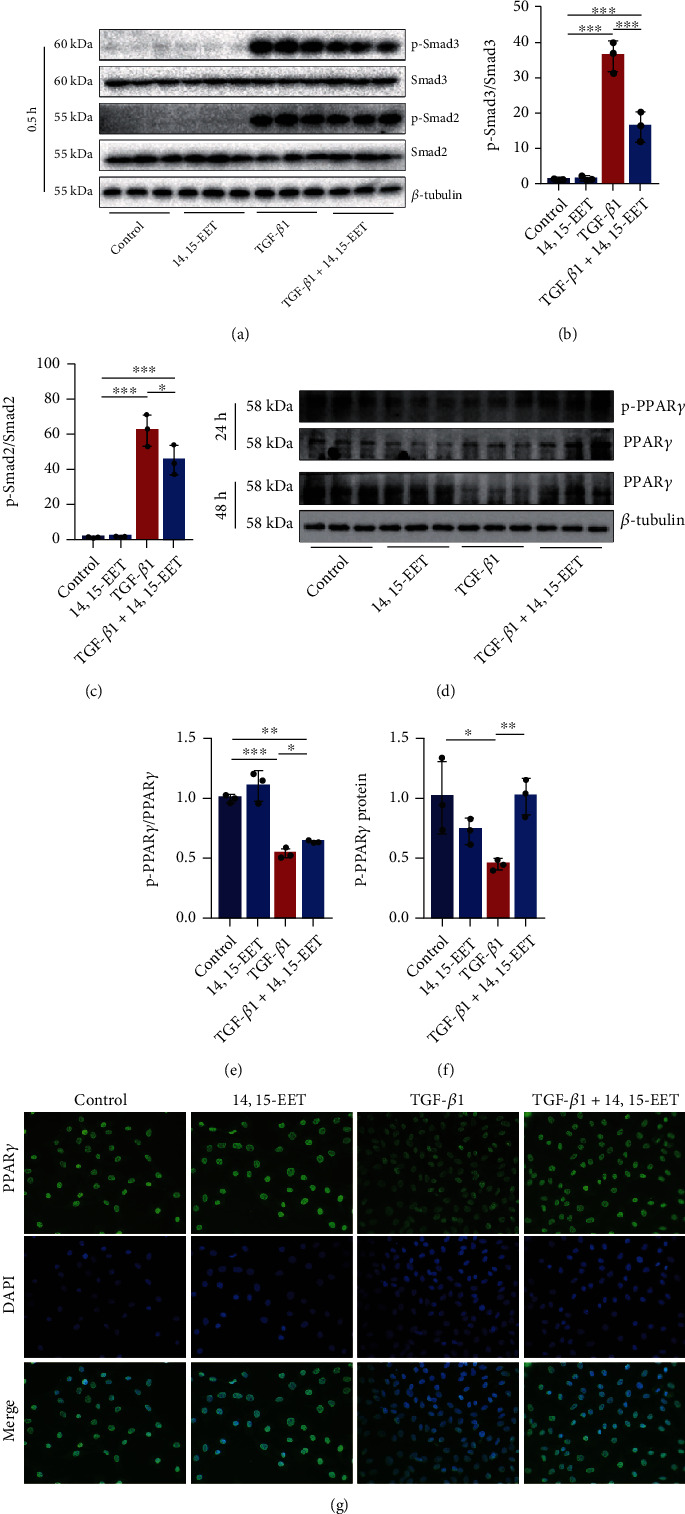
14,15-EET disrupts the TGF-*β*1-Smad2/3 signaling in murine fibroblasts by activating PPAR*γ*. NIH3T3 cells were treated with 14,15-EET (1 *μ*M) for 5 min, followed by TGF-*β*1 treatment (10 ng/mL) for 0.5 h. p-Smad3 and p-Smad2 were detected by western blot ((a)–(c), *n* = 3). NIH3T3 cells were treated with 14,15-EET (1 *μ*M) for 5 min, followed by TGF-*β*1 treatment (10 ng/mL) for 24 h or 48 h. The p-PPAR*γ* and PPAR*γ* were detected by western blot ((d)–(f), *n* = 3). NIH3T3 cells were treated with 14,15-EET (1 *μ*M) for 5 min, followed by treatment with TGF-*β*1 (10 ng/mL) for 48 h. The nuclear location of PPAR*γ* in NIH3T3 cells was detected by immunofluorescence ((g), *n* = 3). ^∗^*P* < 0.05, ^∗∗^*P* < 0.01, and ^∗∗∗^*P* < 0.001.

TGF-*β*1 induces fibroblast proliferation and differentiation by activating the Smad2/3 pathway. We further investigated the mechanism of EETs in inhibiting proliferation and differentiation of TGF-*β*1-treated murine fibroblasts, where 14,15-EET substantially repressed Smad2/3 phosphorylation both of NIH3T3 cells (Figures 5(a)–5(c)) and murine fibroblasts induced by TGF-*β*1 (Figure S2A, B, C). PPAR*γ* is a receptor of EETs [27]. To assess whether EETs downregulated TGF-*β*1 induced the activation of fibroblasts via PPAR*γ*, we quantified PPAR*γ* expression and phosphorylation in NIH3T3 cells and primary murine lung fibroblasts. Western blot showed 14,15-EET restored PPAR*γ* expression of two cell lines stimulated by TGF-*β*1 (Figures 5(d)–5(f), Figure S2D-F). Moreover, treatment with 14,15-EET led to increased phosphorylation of PPAR*γ* in two cell lines compared to the TGF-*β*1 group (Figures 5(d) and 5(e), Figure S2D-E). The immunofluorescence showed that TGF-*β*1 inhibited the fluorescence intensity of nuclear PPAR*γ* of NIH3T3 cells while 14,15-EET significantly increased nuclear PPAR*γ* of the cells treated by TGF-*β*1 (Figure 5(g)). Collectively, these results suggest that 14,15-EET disrupts the TGF-*β*1-Smad2/3 signal in murine fibroblasts by activating PPAR*γ*.

### 3.6. Inhibition of PPAR*γ* Abolishes the Inhibitory Effects of 14,15-EET on the Activation of Murine Fibroblasts

**Figure 6:**
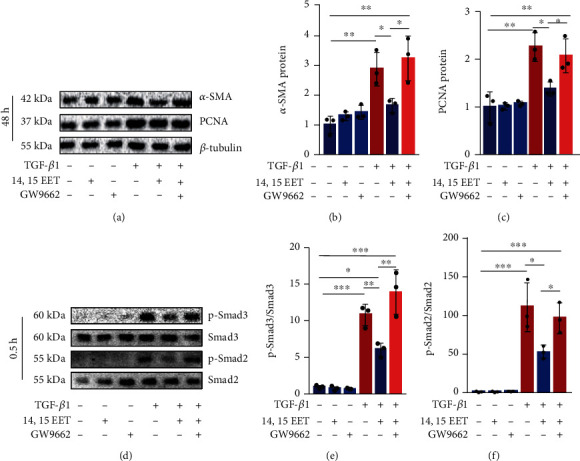
PPAR*γ* inhibitor abolishes the effects of 14,15-EET on the activation of murine fibroblasts. NIH3T3 cells were treated with the GW9662 (10 *μ*M) for 1 h, followed by treatment with 14,15-EET (1 *μ*M) for 5 min and TGF-*β*1 (10 ng/mL) for 48 h. *α*-SMA and PCNA in NIH3T3 cells were detected by western blot ((a)–(c), *n* = 3). Cells were treated with the GW9662 (10 *μ*M) for 1 h, followed by treatment with 14,15-EET (1 *μ*M) for 5 min and TGF-*β*1 (10 ng/mL) for 30 min. p-Smad2 and p-Smad3 were detected by western blot ((d)–(f), *n* = 3). ^∗^*P* < 0.05, ^∗∗^*P* < 0.01, and ^∗∗∗^*P* < 0.001.

Then, we used GW9962, a PPAR*γ* inhibitor, to investigate the role of PPAR*γ* in the inhibitory effects of EETs on fibroblast activation. We found that the inhibitory effects of 14,15-EET were blocked by GW9662 (Figures 6(a)–6(c)). Importantly, GW9662 abolished the reduction of p-Smad2/3 induced by 14,15-EET of TGF-*β*1-treated NIH3T3 cells (Figures 6(d)–6(f)). These data indicate that 14,15-EET inhibits the proliferation and differentiation of fibroblasts via a PPAR*γ*-dependent manner.

## 4. Discussion

**Figure 7:**
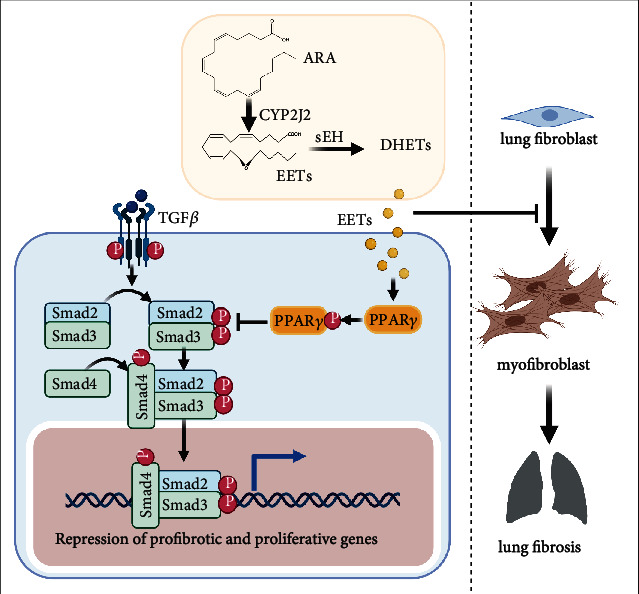
Schematic illustration. EETs inhibit the activation of murine fibroblasts by blocking the TGF-*β*1-Smad2/3 signaling in a PPAR*γ*-dependent manner.

EETs have recently been recognized as critical protective lipid molecules *in vivo*. The metabolic pathway of CYP2J2-EET-sEH plays an essential role in the pathomechanism of fibrosis, while the therapeutic effects of EETs against PF and their specific mechanism are still unclear. In this study, we identified that therapeutical inhibition of EET degradation reversed lung fibrosis in the BLM-induced murine model. Furthermore, we elucidated that the CYP2J2 overexpression and elevating EETs suppressed TGF-*β*1-induced activation of murine fibroblasts, including fibroblast proliferation, differentiation, and collagen secretion by blocking the TGF-*β*1-Smad2/3 signal in a PPAR*γ*-dependent manner (Figure 7). This study suggests that regulating the metabolic pathway of CYP2J2-EETs-sEH is a potential therapeutic strategy in PF.

This study indicates that regulating the metabolic pathway of CYP2J2-EET-sEH attenuates PF by inhibiting the fibroblast activation. A series of changes occur in enzymes involved in EETs production in PF. We have reported that *Cyp2j6* and *Cyp2j9* genes are highly expressed in the lungs of wild-type mice. Both *Cyp2j6* and *Cyp2j9* gene expressions are downregulated in the lungs of BLM-treated mice, while the sEH expression is upregulated [7]. It has been reported that sEH is upregulated in IPF patients [20]. Although our laboratory has shown the prophylactical inhibition of sEH prevented BLM-induced PF in mice [18], neither the therapeutic effect nor the specific mechanism of EETs on PF was elucidated. Our results generally illustrate the potential therapeutic role of the CYP2J2-EET-sEH pathway in chronic lung disease. We further showed the therapeutic effect of EETs on PF. The treatment with TPPU on the 7^th^ day reversed lung fibrosis in BLM-treated mice by inhibiting the activation of fibroblasts. He et al. found that 11,12-EET attenuates fibroblast activation in Ang II-induced cardiac fibrotic response in mice by targeting G*α* 12/13 [28]. Moreover, we found the role of EETs on the activation of fibroblasts is PPAR*γ*-dependent.

As the primary effector cells of fibrosis, myofibroblasts actively interact with surrounding cells in the fibrotic microenvironment. On the one hand, myofibroblasts lead to epithelial cell damage in PF, which results in the activation of fibroblasts. On the other hand, myofibroblasts could be activated by changing the microenvironment of fibrosis [29]. We demonstrated that inhibition of sEH reduced *α*-SMA in the lungs of BLM-induced mice. *In vitro*, we also confirmed that CYP2J2 overexpression and EETs inhibited proliferation and differentiation of murine fibroblasts induced by TGF-*β*1. A similar effect of EETs has been observed in cardiac fibrosis [28], which confirms our findings. Myofibroblasts secrete various cytokines to promote epithelial cell damage, then aggravate fibroblast-myofibroblast transformation, and eventually produce a large number of myofibroblasts. The interaction between myofibroblasts and the fibrotic microenvironment leads to a vicious loop, eventually forming nonfunctional fibrotic tissue [29]. The overexpression of CYP2J2, elevating EETs, and inhibition of sEH successfully attenuate TGF-*β*1-induced activation of murine fibroblasts.

Our work highlights the importance of the EETs-PPAR*γ* axis to downregulate detrimental TGF-*β*1 pathways by inactivating the proliferation and differentiation of lung fibroblasts. PPAR*γ*, a member of the ligand-activated transcription factor superfamily, expresses in many organs, such as the lung. PPAR*γ* involves gene expression, glucose metabolism, lipid metabolism, fibrosis, inflammation, and oxidative stress [30, 31]. PPAR*γ* could be activated by phosphorylating to exert biological effects [32]. It has been reported that the activation of PPAR*γ* plays a protective role by inhibiting TGF-*β*1-target gene expression in mice [33]. We observed that 14,15-EET promoted PPAR*γ* expression and phosphorylation in murine fibroblasts treated by TGF-*β*1. Besides, 14,15-EET restored nuclear translocation of PPAR*γ* in the fibroblasts stimulated by TGF-*β*1. TGF-*β*1-Smad2/3 signal is a classic pathway in the process of PF. Our study shows that EETs inhibited the phosphorylation of Smad2/3 by activating PPAR*γ* at the molecular level. In line with our results, activating the PPAR*γ* inhibited TGF-*β*1-induced Smad2/3 phosphorylation [34, 35]. PPAR*γ* directly binding to Smad3 plays a protective role, which has been reported in pulmonary arterial hypertension (PAH) [24].

However, the specific mechanism by which PPAR*γ* activated by EETs inhibits Smad2/3 phosphorylation in lung fibroblasts is unclear. It has been reported that activated PPAR*γ* directly binds to Smad3 in VSMCs, reversing PAH [24], which confirms our findings. Moreover, we showed that all types of EETs could inhibit the activation of murine fibroblasts induced by TGF-*β*1, but only 14,15-EET was used in the following experiments. Because 14,15-EET abounds in the lungs [36], CCK8 assay showed the most significant inhibitory effect on proliferation compared to the other three types of EETs.

In conclusion, our study shows the essential therapeutically antifibrotic role of EETs in PF. We reveal that EETs block fibroblast activation through the PPAR*γ* pathway, contributing to the therapeutic effect of EETs against PF in mice. Regulating CYP2J2-EET-sEH metabolic pathway may be a potential therapeutic option for PF and chronic lung diseases.

## Figures and Tables

**Table 1:** Sequences of specific primers used in this study.

Gene	Forward primer (5′-3′)	Reverse primer (5′-3′)
h-*CYP2J2*	TGCGATGGGCTCTGCTTTAT	GGATCATGGTACCCTTGGGC
m-*Gapdh*	AAGAGGGATGCTGCCCTTAC	CTCGTGGTTCACACCCATCA

## Data Availability

All data included in this study are available upon request by contact with the corresponding authors.

## Supplementary Materials

Supplementary Materials Figure S1: 14,15-EET attenuates TGF-*β*1-induced activation of primary murine lung fibroblasts. The primary murine lung fibroblasts were treated with 14,15-EET (1 *μ*M) for 5 min, followed by treatment with TGF-*β*1 (5 ng/mL) for 48 h. The cell proliferation was examined by CCK-8 ((a), *n* = 5). Protein levels of COL-I, *α*-SMA, and PCNA were detected by western blot ((b)–(e), *n* = 3). ^∗^*P* < 0.05, ^∗∗^*P* < 0.01, and ^∗∗∗^*P* < 0.001. Figure S2: 14,15-EET upregulates PPAR*γ* and downregulates the Smad2/3 signaling of TGF-*β*1-induced primary murine lung fibroblasts. The primary murine lung fibroblasts were treated with 14,15-EET (1 *μ*M) for 5 min, followed by treatment with TGF-*β*1 (5 ng/mL) for 0.5 h. p-Smad3 and p-Smad2 were detected by western blot ((a)–(c), *n* = 3). The primary murine lung fibroblasts were treated with 14,15-EET (1 *μ*M) for 5 min, followed by treatment with TGF-*β*1 (5 ng/mL) for 24 h or 48 h. The p-PPAR*γ* and PPAR*γ* were detected by western blot ((d)–(f), *n* = 3). ^∗^*P* < 0.05, ^∗∗^*P* < 0.01, and ^∗∗∗^*P* < 0.001.
