# Supplementary material for: Epoxyeicosatrienoic Acids Inhibit the Activation of Murine Fibroblasts by Blocking the TGF-β1-Smad2/3 Signaling in a PPARγ-Dependent Manner
Source: Oxid Med Cell Longev. 2022 Oct 13;2022:7265486. doi: 10.1155/2022/7265486 (PMC9584742; doi:10.1155/2022/7265486)
Supplement: Supplementary Materials — Figure S1: 14,15-EET attenuates TGF-β1-induced activation of primary murine lung fibroblasts. The primary murine lung fibroblasts were treated with 14,15-EET (1 μM) for 5 min, followed by treatment with TGF-β1 (5 ng/mL) for 48 h. The cell proliferation was examined by CCK-8 ((a), n = 5). Protein levels of COL-I, α-SMA, and PCNA were detected by western blot ((b)–(e), n = 3). ∗P < 0.05, ∗∗P < 0.01, and ∗∗∗P < 0.001. Figure S2: 14,15-EET upregulates PPARγ and downregulates the Smad2/3 signaling of TGF-β1-induced primary murine lung fibroblasts. The primary murine lung fibroblasts were treated with 14,15-EET (1 μM) for 5 min, followed by treatment with TGF-β1 (5 ng/mL) for 0.5 h. p-Smad3 and p-Smad2 were detected by western blot ((a)–(c), n = 3). The primary murine lung fibroblasts were treated with 14,15-EET (1 μM) for 5 min, followed by treatment with TGF-β1 (5 ng/mL) for 24 h or 48 h. The p-PPARγ and PPARγ were detected by western blot ((d)–(f), n = 3). ∗P < 0.05, ∗∗P < 0.01, and ∗∗∗P < 0.001. [file 7265486.f1.pdf]

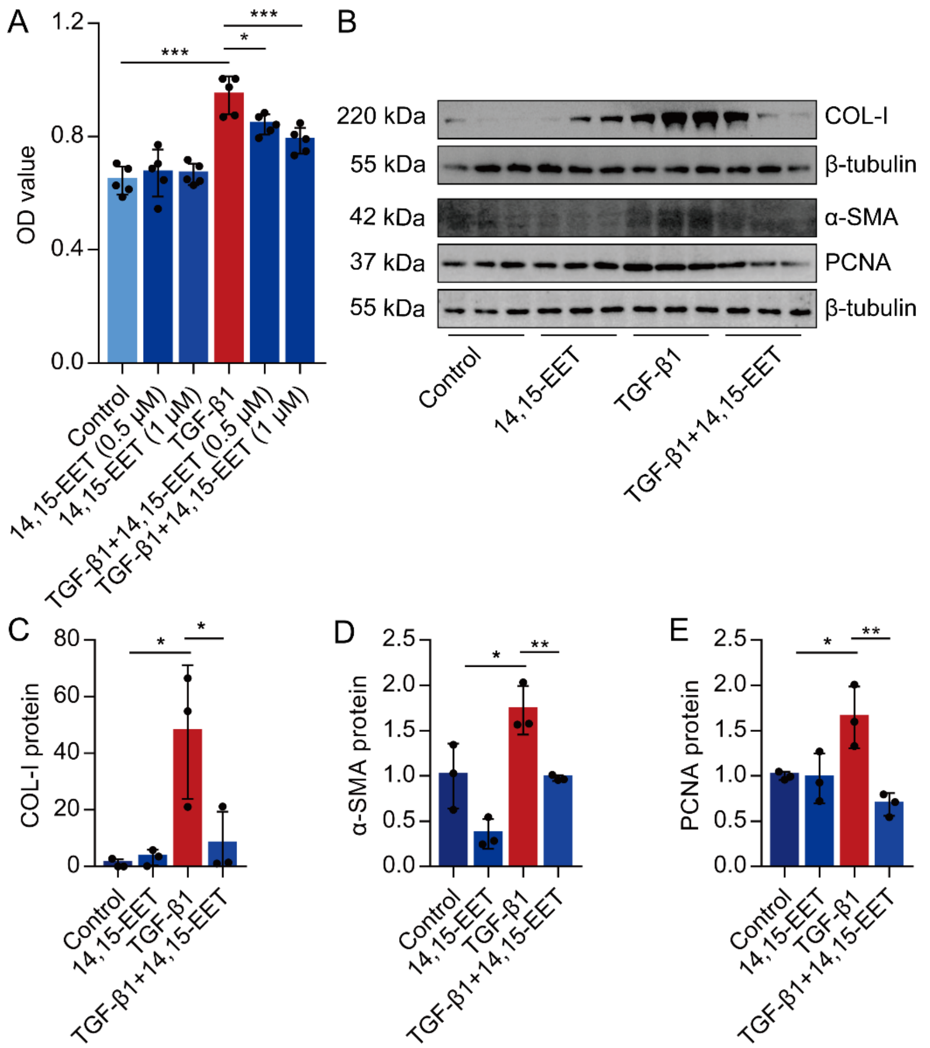

Figure S1. 14,15-EET attenuates TGF-β1-induced activation of primary murine lung fibroblasts. The primary murine lung fibroblasts were treated with 14,15-EET (1 μM) for 5 min, followed by treatment with TGF-β1 (5 ng/mL) for 48 h. The cell proliferation was examined by CCK-8 (A, *n* = 5). Protein levels of COL-I, α-SMA, and PCNA were detected by western blot (B-E, *n* = 3). **P* < 0.05, ***P* < 0.01, and ****P* < 0.001.

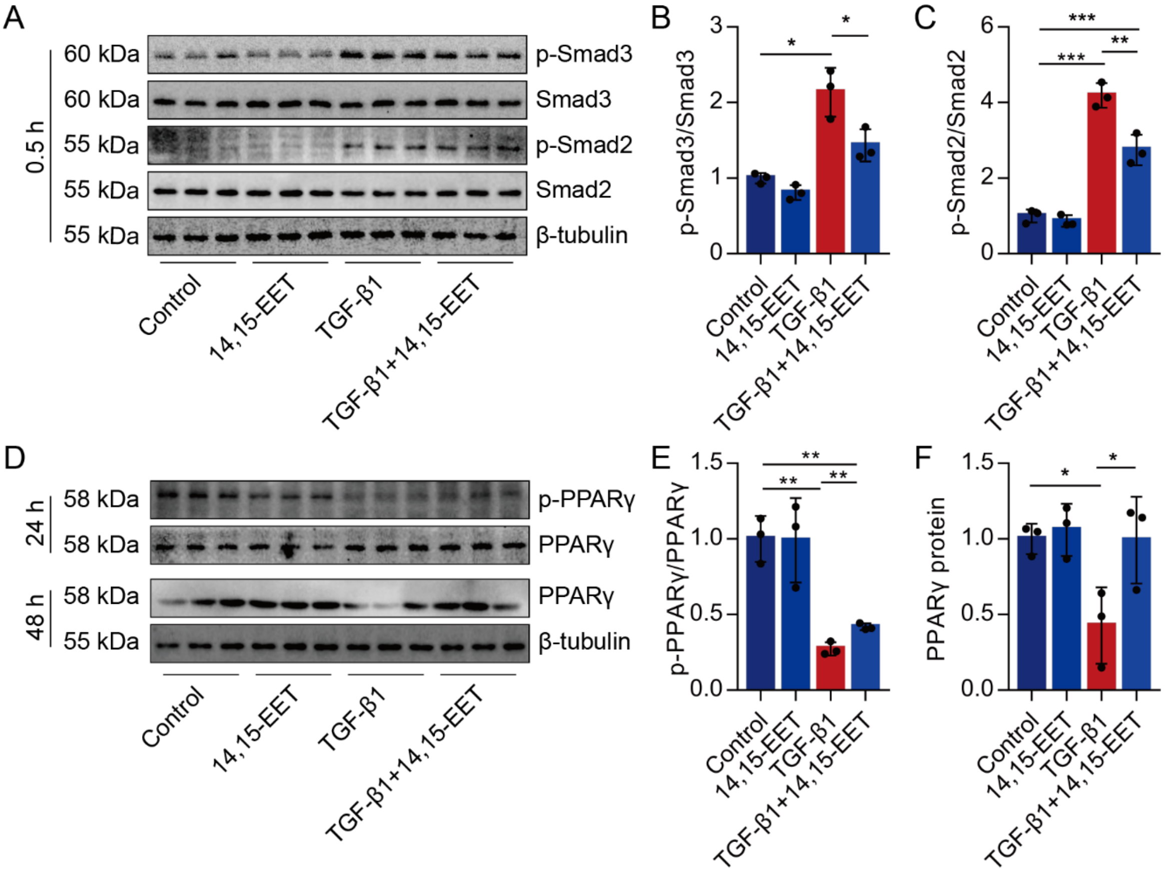

Figure S2. 14,15-EET up-regulates PPARγ and down-regulates the Smad2/3 signaling of TGF-β1-induced primary murine lung fibroblasts. The primary murine lung fibroblasts were treated with 14,15-EET (1 μM) for 5 min, followed by treatment with TGF-β1 (5 ng/mL) for 0.5 h. p-Smad3 and p-Smad2 were detected by western blot (A-C, $n = 3$). The primary murine lung fibroblasts were treated with 14,15-EET (1 μM) for 5 min, followed by treatment with TGF-β1 (5 ng/mL) for 24 h or 48 h. The p-PPARγ and PPARγ were detected by western blot (D-F, $n = 3$). *$P < 0.05$, **$P < 0.01$, and ***$P < 0.001$.
